# Docosahexaenoic acid ameliorates autistic‐like behaviors by inhibiting oxidative stress and inflammatory response in neonatal maternal separation rats

**DOI:** 10.1002/pdi3.91

**Published:** 2024-06-19

**Authors:** Boqing Xu, Hao Yuan, Xiaohuan Li, Qingyang Yu, Chunfang Dai, Zhifang Dong

**Affiliations:** ^1^ Growth, Development, and Mental Health of Children and Adolescence Center Pediatric Research Institute Ministry of Education Key Laboratory of Child Development and Disorders National Clinical Research Center for Child Health and Disorders Chongqing Key Laboratory of Child Neurodevelopment and Cognitive Disorders Children's Hospital of Chongqing Medical University Chongqing China; ^2^ Department of Children Health Care Guangzhou Women and Children's Medical Center Guangzhou Medical University Guangzhou Guangdong China

**Keywords:** autism spectrum disorder, docosahexaenoic acid, inflammatory response, JNK, oxidative stress

## Abstract

Autism spectrum disorder (ASD) is a pervasive neurodevelopmental disorder characterized by impaired social interactions and communication, repetitive, or stereotyped behavior. Docosahexaenoic acid (DHA), an essential polyunsaturated fatty acid, has been demonstrated to exert anti‐oxidative stress and anti‐inflammatory properties, while also promoting myelin development and neural differentiation and development. However, it remains uncertain whether DHA can ameliorate autistic‐like behaviors, and if so, the underlying mechanisms are still unclear. Here, we established a neonatal maternal separation (NMS) rat model and treated it with DHA (80 mg/kg/day, i.p.). The results showed DHA treatment significantly alleviated autism‐like behaviors in the NMS rats during their juvenile period. Subsequently, we employed network pharmacology analysis and molecular docking methods to screen potential targets of DHA in ASD therapy. Through Gene Ontology, Kyoto Encyclopedia of Genes and Genomes, and Wikipedia enrichment analysis, we identified anti‐oxidative stress, anti‐inflammatory and JNK signaling pathway that might be associated with DHA‐mediated improvement of autistic‐like behaviors. Furthermore, western blotting assays showed DHA significantly downregulated the expression levels of p‐JNK and c‐JUN, while upregulating the expression levels of NRF2, HO‐1, SOD1, and CAT. In addition, enzyme‐linked immunosorbent assay results showed DHA effectively reduced the production of pro‐inflammatory factors, such as TNF‐α, IL‐6, and IL‐1β. Collectively, our study predicted and validated that DHA exhibits the potential to improve autistic‐like behaviors induced by NMS in rats by suppressing JNK activation and inhibiting oxidative stress and inflammatory response. These findings suggest that DHA may be a potential therapeutic agent for the treatment of ASD.

## Graphical abstract

The JNK pathway and oxidative stress are activated, and pro‐inflammatory cytokines are increased in neonatal maternal separation (NMS) rats. While docosahexaenoic acid treatment suppresses the activation of JNK pathway and oxidative stress, and reduces pro‐inflammatory cytokines release, thereby improving autism‐like behavior induced by NMS.

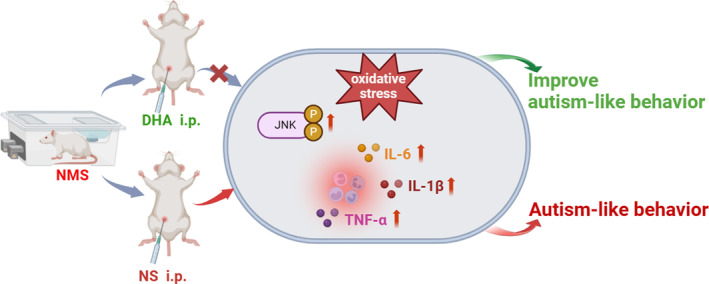

## 1 INTRODUCTION

Autism spectrum disorder (ASD) is a pervasive neurodevelopmental disorder characterized by impaired social interactions and communication, repetitive, or stereotyped behavior. It is one of the fastest‐growing disabilities in terms of patient numbers, placing enormous mental burden and financial strain on families and society. Unfortunately, no scientific studies have confirmed that ASD can be completely and effectively cured so far. The critical period of brain development occurs within the first 1–2 years of life, with the brain weighing up to 3/4 of a person's body weight by the age of 2. Brain tissue then develops relatively slowly through adolescence, with the development of the prefrontal cortex (PFC) continuing into adulthood.^1^ Studies have shown that premature separation of infants from their mothers, adverse environmental stimuli, nutritional deficiencies, and other stimuli in the early stage of human life can induce disorders such as ASD.^2^, ^3^ Since a variety of factors are involved in the pathogenesis of ASD, such as genetic factors, immunity, redox imbalance, and chronic inflammation, among other doctrines, these complex pathophysiologic mechanisms of ASD have led to the lack of its effective treatment.^4^, ^5^

Children have a low level of resistance to oxidative stress and are susceptible to disorders caused by external environmental influences, such as irritation, infection, injury, and so on.^6^ Abnormal oxidative stress is the common driver of the pathogenesis of neurological diseases such as ASD. Many reports show that children with autism have elevated markers of oxidative stress in their brains and peripheral circulation and that these levels are correlated with the severity of their condition.^7^, ^8^, ^9^ Research studies have shown that antioxidant supplementation can be helpful for some children with autism.^10^ The inflammatory response exhibits a close association with oxidative stress, often resulting in aberrant mitochondrial function, and the induction of oxidative stress in the presence of inflammation.^11^ Furthermore, numerous predisposing genes implicated in ASD also serve to regulate immune function, and aberrations within the peripheral immune system following infection contribute to an elevation in pro‐inflammatory cytokines.^12^, ^13^ It has been found that microglia can be activated and a significant increase in inflammatory cytokines, such as tumor necrosis factor‐α (TNF‐α) and interleukin‐6 (IL‐6), can be detected in the brains of ASD patients.^14^

Docosahexaenoic acid (DHA), a crucial polyunsaturated fatty acid, contributes significantly to the structure and functionality of the cytomembrane while playing a pivotal role in various aspects such as the development of the central nervous system and mental capacity, vision enhancement, sleep quality improvement, and better circulation.^15^, ^16^, ^17^, ^18^ For children who suffer from developmental and behavioral disorders associated with low blood concentrations of polyunsaturated fatty acids, supplementation with a range of fatty acids, including DHA, can be advantageous in enhancing their learning abilities and behavioral outcomes.^19^ Recent studies have proposed that DHA has obvious potential in regulating immunity, anti‐oxidative stress, and anti‐inflammation, as well as promoting neural differentiation and development, and myelin sheath development.^20^, ^21^, ^22^, ^23^ Of particular concern is the fact that the amount of DHA in the brain increases dramatically from late pregnancy to early infancy, which is not only the period when the brain's nerves and synapses are most actively developing but also the period when the brain is most susceptible to the influence of the external environment.^24^ In addition, DHA is metabolized in living organisms to produce active substances that also have neuroprotective, neurotrophic, and antioxidant effects.^25^, ^26^ However, it is currently unclear whether administration of DHA makes a difference in improving neural development in ASD, and if so, the underlying mechanisms remain unclear.

Network pharmacology is an emerging strategy for constructing biomedical interaction networks to assess drug molecular mechanisms.^27^ It explains the scientific difficulties in pharmacological research by studying and systematizing multiple dimensions of a target.^28^ The qualities, such as more complex compositions, multiple targets, and involvement of a wide range of signaling pathways, make the study of compounds challenging. Therefore, more and more studies have applied the network pharmacology approach to explore and visualize the links between drugs, targets, and diseases.^29^, ^30^ Molecular docking offers an intensive explanation of intermolecular interactions and visually depicts the mechanism underlying these interactions, thereby playing a crucial role in elucidating drug functionalities. In this study, we first observed the effect of DHA on autism‐like behavior in neonatal maternal separation (NMS) rats, one of the animal models of ASD. Then, we used a network pharmacology approach to obtain the common targets of DHA and ASD by searching different databases, and protein–protein interaction (PPI) network analysis. Moreover, molecular docking was used to confirm the prediction and in vivo experiments are conducted to verify the main targets and pathways.

## 2 MATERIALS AND METHODS

### 2.1 Experimental animals

**FIGURE 1:**
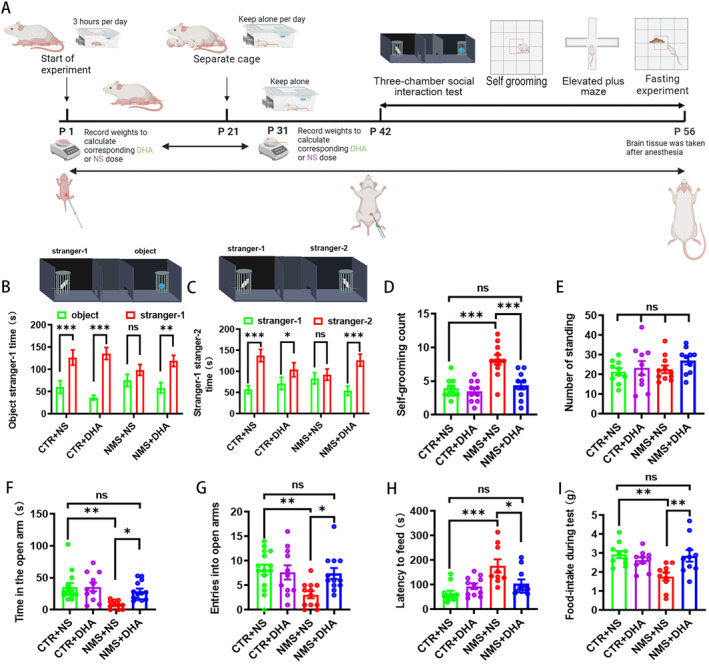
DHA ameliorates autistic‐like behaviors induced by NMS. (A) The experiment timeline. (B) DHA treatment significantly increased the time spent with stranger‐1 rat in NMS rats in the first phase of the three‐chamber sociability test. (C) DHA treatment significantly increased the time spent with stranger‐2 rat in NMS rats in the second phase of the three‐chamber sociability test. (D) DHA treatment significantly decreased the number of repetitive self‐grooming induced by NMS in the open field test. (E) DHA treatment has no effect on the number of times the rats stood upright in NMS rats in the open field test. (F) DHA treatment fully increased the time spent in the open arm in NMS rats during the elevated plus maze test. (G) DHA treatment fully increased the number of entering into the open arms in NMS rats during the elevated plus maze test. (H) DHA treatment significantly shortened the feeding latency in NMS rats during the fasting experiment. (I) DHA treatment significantly increased the amount of food consumed during the experimental period in NMS rats in the fasting experiment. The results were presented as the mean ± SEM. DHA, docosahexaenoic acid; NMS, neonatal maternal separation. *p < 0.05, **p < 0.01, ***p < 0.001.

Female and male Sprague–Dawley (SD) rats (purchased from the experimental animal center of Chongqing Medical University) were mated at the experimental animal center of Children's Hospital of Chongqing Medical University, and neonatal male pups were used for experimental studies. Pregnant females were individually housed in plastic cages in temperature‐controlled (21°C) colony chambers on a 12/12 h light/dark cycle (8 a.m. to 8 p.m.). Food and water were available ad libitum. The establishment of the NMS rat model was divided into two phases: (1) the NMS phase: from postnatal day 1 to day 21, mothers were separated from their infants for 3 h per day. (2) The isolation rearing phase: rats experiencing NMS were reared in single cages from day 22, until day 56. Behavioral tests were performed during postnatal day 42 to day 56. The experimental timeline is shown in Figure 1A. In the present study, we used a total of 14 adult rats for breeding purposes and obtained about 63 male rat pups from these pairings. All procedures were performed following the guidelines for animal research of the Chongqing Municipal Science and Technology Commission and approved by the Animal Ethics Committee of Children's Hospital of Chongqing Medical University (No.CHCMU‐IACUC20220323015).

### 2.2 Antibodies and reagents

DHA (#DH105‐2) was purchased from Selleck Chemicals LLC (Houston, TX, USA). Rabbit anti‐p‐JNK (#80024‐1‐RR), rabbit anti‐JNK (#17572‐1‐AP), rabbit anti‐c‐JUN (#24909‐1‐AP), rabbit anti‐NRF2 (#80593‐1‐RR), rabbit anti‐HO‐1 (#10701‐1‐AP), rabbit anti‐SOD1 (#10269‐1‐AP) and rabbit anti‐CAT (#21260‐1‐AP) were obtained from Proteintech (Chicago, IL, USA). Mouse anti‐β‐actin (#HC201‐02) was supplied by TransGen Biotech (Beijing, China). Mouse anti‐GAPDH (#6004‐1‐1g) was obtained from Proteintech (Chicago, IL, USA). Bicinchoninic acid (BCA) Kit was obtained from Thermo Fisher Scientific (Waltham, Massachusetts, USA). Rat TNF‐α enzyme‐linked immunosorbent assay (ELISA) Kit (#JL13202‐96T), rat IL‐6 ELISA Kit (#JL20896‐96T) and rat IL‐1β ELISA Kit (#JL20884‐96T) were purchased from Jianglai Biotechnology (Shanghai, China). Radio immunoprecipitation assay (RIPA) lysis buffer (#P0013B) was obtained from Beyotime Biotechnology (Shanghai, China).

### 2.3 DHA treatment

Each litter of neonatal male rats was randomly divided into control (CTR) + normal saline (NS), CTR + DHA, NMS + NS, and NMS + DHA groups. DHA was dissolved in dimethyl sulfoxide (DMSO) and then diluted in NS. The rats in CTR + DHA and NMS + DHA groups received intraperitoneal (i.p.) injections of DHA at a dose of 80 mg/kg, once a day for up to 56 days, while the rats in CTR + NS and NMS + NS groups were injected intraperitoneally with the same dose of DMSO + NS.^31^ The specific method is shown in Figure 1A.

### 2.4 Behavioral experiments

1. Three‐chamber social interaction test: The social interaction experiment was performed in a three‐chamber animal social behavior test apparatus (60 × 40 × 20 cm). This setup was evenly partitioned into three sections, interconnected, and comprised of an object chamber and a companion chamber at opposing ends, with an empty chamber situated in the middle. Object and an unfamiliar male SD rat of the same age were placed in the same cage in the corners corresponding to the object room and the companion room, respectively. On the day before the formal experiment, the test rats were placed in the boxes for 5 min each. When the formal experiment started, the experimental rats were placed in the middle empty chamber, ensuring that each rat's head orientation and position were consistent, and the entire experiment lasted for 5 min. The times at which the rats entered the object room and the companion room, respectively, were recorded by ANY‐maze animal behavior software (Stoelting, USA) to evaluate the social interaction behavior of the test rats. The experiment was conducted in a quiet environment and the experimental equipment was cleaned with 75% alcohol after the completion of the experiment for each animal.
2. Open field test: Open field test setup (dimensions of 60 × 60 × 60 cm) was used to detect self‐grooming behaviors in rats. One day before the experiment, the rats were placed in the device to acclimatize them to the environment. At the time of the experiment, the experimental rats were positioned at the device and their activities were recorded for 5 min using ANY maze animal behavior software (Stoelting, USA). Self‐grooming behavior and the number of times they stood upright during this time were counted to evaluate rats' self‐grooming behaviors. The experimental equipment was cleaned with 75% alcohol between tests.
3. Elevated plus maze test: The maze consists of two open and closed arms (with a 40 cm high wall on the closed arm) aligned at right angles (50 cm per arm). In the experiment, the experimental rats were positioned at the device (10 × 10 cm) and allowed to explore it for 5 min at a height of 50 cm above the floor. The number of entries and time spent in the open arm were recorded using ANY‐maze animal behavior software (Stoelting, USA). The device was cleaned with 75% alcohol between tests.
4. Fasting experiment: Prior to the experiment, the rats were deprived of food for 48 h. Subsequently, they were placed in the experimental testing room and given 1 h to adapt to the surroundings. Next, the open field test setup was used to detect the anxiety state of the rats. Before the start of the experiment, the food chow which had been weighed in advance was placed in the center of the open field. After the experiment started, we observed and recorded the time the experimental animals first ate within 12 min and the amount of food they ate. Afterward, the experimental rats were placed in the cage with the pre‐weighed feed and continued to observe and record their food intake for the following 30 min to guarantee the comprehensiveness and precision of the data.

### 2.5 Prediction of drug targets for DHA

We used Symptom Mapping (http://www.symmap.org/. Accessed 01 Oct 2023) with “Docosahexaenoic acid” as the keyword to obtain the target genes of compound DHA in the SymMap database,^32^ and created a component‐target gene network using Cytoscape v3.9.1 program (https://cytoscape.org/. Accessed 2 Oct 2023) to create the component‐target gene network.^33^

### 2.6 Prediction of targets for ASD

Potential ASD‐related targets were retrieved from the Human Genome Database (GeneCards, https://www.genecards.org/. Accessed 2 Oct 2023)^34^ and the DisGeNET database (https://www.disgenet.org/. Accessed 2 Oct 2023) to search for potential ASD‐related targets.^35^

### 2.7 Network construction and enrichment analysis

The screened DHA targets and targets of ASD‐associated proteins were imported into the VENNY2.1 web tool (https://bioinfogp.cnb.csic.es/tools/venny/. Accessed 2 Oct 2023) to be analyzed for potential targets of DHA action on ASD. They were uploaded into the search tool designed to retrieve interacting genes/proteins from the database (STRING; https://cn.string‐db.org/. Accessed 2 Oct 2023), enabling the construction of a PPI network specifically for the DHA related to ASD.^36^ We set the STRING parameters with medium confidence (0.4), rattus norvegicus, network nodes represent genes/proteins, and connectors represent protein–protein associations.^37^ We used DAVID (https://david.ncifcrf.gov/summary.jsp. Accessed 2 Oct 2023) for Kyoto Encyclopedia of Genes and Genomes (KEGG) pathway analysis and Wikipedia enrichment analysis,^38^ and Hiplot (https://hiplot.com.cn/. Accessed 2 Oct 2023) for Gene Ontology (GO) enrichment analysis, KEGG enrichment analysis and visualization of the results,^39^ with a selection of *p* < 0.05. GO enrichment was mainly used to examine the targets' biological processes (BP), cellular components (CC) and molecular functions (MF). KEGG pathway enrichment was used to examine the key biological pathways of the targets.

### 2.8 Molecular docking analysis

Through GO and KEGG pathway enrichment analysis, we identified key gene targets of DHA. These targets were confirmed by molecular docking with DHA.

We retrieved the corresponding MOL2 formula (PDB format) from the Research Collaboratory for Structural Bioinformatics (RCSB) database (https://www.rcsb.org Accessed 3 Oct 2023)^40^ and the AlphaFold protein structure database (https://alphafold.com. Accessed 3 Oct 2023).^41^ Utilizing the RCSB database, the molecular structures of the core genes were determined. Subsequently, these structures were edited using Pymol software (https://pymol.org/. Accessed 10 Oct 2023),^42^ involving the removal of solvents and organic components. AutoDockTools software was used to add hydrogen atoms before molecular docking. The active ingredients were used as ligands and the core genes as receptors. Molecular docking was performed using AutoDock Vina.^43^ The software predicts the binding energy value, potential conformation, bond distance, and interaction type of the docked molecules using the standard docking procedure, “Number of GA Runs”, as per the official recommendation of 50 runs and other default parameters automatically. PyMOL software is finally used to display the results. The molecular docking results were chosen based on their highest affinity and lowest binding energy.

### 2.9 Western blotting

After completion of behavioral studies, rats were deeply anesthetized with ethyl carbamate and then rapidly decapitated after transcardiac perfusion of phosphate‐buffered saline (PBS). Prefrontal tissue samples were immediately collected for western blotting. For total protein extraction, brain tissue was homogenized in a 1.0 mL cold RIPA lysis buffer containing a mixture of protease inhibitors (Complete, Roche, Basel, Switzerland), and then centrifuged at 12,000 rpm for 15 min at 4°C to collect the supernatant. Protein concentration was measured using a BCA assay (Thermo Fisher Scientific, Waltham, USA). Equivalent proteins were denatured in 5 × loading buffer for 5 min at 98°C. Protein samples were loaded on a 10% Sodium dodecyl sulfate‐polyacrylamide gel electrophoresis (SDS‐PAGE) gel for electrophoresis and then transferred to a polyvinylidene fluoride (PVDF) membrane. The membranes were incubated at room temperature for 30 min using an efficient western rapid‐blocking solution (Genefist, #GF1815, Shanghai, China) and then incubated with primary antibodies (p‐JNK, JNK, c‐JUN, NRF2, HO‐1, SOD1, CAT) overnight in a shaker at 4°C. On the second day, all membranes were washed three times with PBST for 5 min each and incubated with corresponding horseradish peroxidase (HRP)‐conjugated secondary antibody (1:3000, Thermo Fisher Scientific, Waltham, MA, USA) for 90 min at room temperature. The protein was detected using a Bio‐Rad Imager (Bio‐Rad, Hercule, CA, USA) with enhanced chemiluminescence (ECL) western blotting substrate (Pierce, Waltham, USA). The Bio‐Rad Quantity One software was used to quantify the band intensity of each protein. *β*‐actin or GAPDH was used as an internal control.

### 2.10 Enzyme‐linked immunosorbent assay

After completion of behavioral studies, rats were deeply anesthetized and then transcardially perfused with PBS. The brains of rats in each group were taken and underwent 2 freeze‐thaw cycles to break the cellular membranes. These tissues were then homogenized in ice‐cold conditions, maintaining a tissue‐to‐saline ratio of 1:9. Lysates were centrifuged at 5000 g for 5 min at 4°C. The supernatant was collected and the concentrations of IL‐6, interleukin‐1β (IL‐1β) and TNF‐α were measured using an ELISA Kit according to the manufacturer's instructions. Absorbance was measured at 450 nm using a Bio Tek Instruments enzyme marker, and the concentration of the target proteins was then computed based on the standard curve and normalized relative to the protein content of the samples.

### 2.11 Statistical analysis

All data were expressed as means ± standard error (mean ± SEM). A two‐tailed *t*‐test was used to evaluate the statistical significance between the two groups. Information from multiple groups was analyzed using one‐way analysis of variance (ANOVA) or repeated measures ANOVA followed by Tukey's post hoc test. Statistical significance was *p < 0.05, **p < 0.01, ***p < 0.001; ns, no significantly different.

## 3 RESULTS

### 3.1 DHA rescued social deficits in NMS rats

Social interaction and communication disorders are core symptoms of autism. To determine whether DHA improved the social deficits induced by NMS, we first tested social interaction. As shown in Figure 1, in the first phase, there were no notable disparities in the amount of time spent by the NMS + NS group rats between interacting with stranger‐1 rat and the object (NMS + NS: *n* = 12; Figure 1B). In the second phase, there was likewise no significant difference in the time of NMS + NS group rats communicated with stranger‐1 rat and the previously unseen stranger‐2 rat (NMS + NS: *n* = 12; Figure 1C). These results indicat that NMS impairs social interaction behavior in rats, suggesting abnormal brain development in neonatal rats after NMS. Interestingly, administration of the DHA obviously rescued the social deficits, as reflected by, in the first phase of the experiment. We observed that rats in the NMS + DHA group spent visibly more time with stranger‐1 rat than the object (NMS + DHA: *n* = 16; Figure 1B). Also in the second phase, rats in the NMS + NS group were significantly more inclined to communicate with stranger‐2 rat, whom they had not met before (NMS + DHA: *n* = 16; Figure 1C). Taken together, these results indicate that DHA administration can overcome the social deficits induced by NMS.

### 3.2 DHA improves repetitive self‐grooming behavior in NMS rats

In rodents, repetitive self‐grooming behavior reflects the characteristics of repetitive stereotype‐like behavior and thus can be used as an indicator to detect repetitive or stereotyped behavior in ASD. As shown in Figure 1, compared with the CTR + NS group rats, the number of repetitive self‐grooming in NMS + NS group rats was significantly increased (CTR + NS: *n* = 10; NMS + NS: *n* = 11, *p* < 0.001 vs. CTR + NS; Figure 1D), indicating a significant repetitive stereotype‐like behavior in rats who underwent NMS. Interestingly, treatment with DHA significantly improved the repetitive stereotype‐like behavior, as reflected by an obvious decrease in the number of repetitive self‐grooming (NMS + DHA: *n* = 10, *p* < 0.001 vs. NMS + NS; Figure 1D), whereas DHA treatment had no effect on the number of repetitive self‐grooming in the CTR group (CTR + DHA: *n* = 10, *p* > 0.05 vs. CTR + NS; Figure 1D). In addition, we also observed the number of times the rats stood upright as a manifestation of curiosity about the external environment, but no notable disparities were found between the four groups (Figure 1E). This experiment suggests that NMS increases repetitive stereotype‐like behavior in rats, and this behavior can be improved by DHA treatment.

### 3.3 DHA ameliorates anxiety‐like behavior in NMS rats

Anxiety is the most common co‐occurring disorder in children on the autism spectrum, and to confirm whether DHA can improve anxiety‐like behavior in NMS rats, the elevated plus maze test was conducted. As shown in Figure 1, in comparison to the control rats, the time spent exploring the open arms and the number of entering into the open arms in NMS + NS group rats were significantly reduced (CTR + NS: *n* = 14; NMS + NS: *n* = 11, *p* < 0.001 vs. CTR + NS; Figure 1F,G), indicating a significant anxiety‐like behavior in rats suffered NMS. Whereas treatment with DHA fully ameliorated the NMS‐induced anxiety‐like behavior, as reflected by a dramatic increase in the time spent exploring the open arms (NMS + DHA: *n* = 13, *p* < 0.05 vs. NMS + NS; Figure 1F) and the number of entering into the open arms (NMS + DHA: *n* = 13, *p* < 0.05 vs. NMS + NS; Figure 1G).

To further evaluate whether DHA can improve anxiety‐like behavior induced by NMS, we introduced another behavioral model of anxiety under laboratory conditions, the fasting experiment. The result showed that rats in the NMS + NS group had a significantly longer eating latency compared with rats in the CTR + NS group (CTR + NS: *n* = 10; NMS + NS: *n* = 10, *p* < 0.001; Figure 1H), and rats in the NMS + NS group ate significantly less food compared with rats in the CTR + NS group (CTR + NS: *n* = 10; NMS + NS: *n* = 10, *p* < 0.01; Figure 1I). As expected, treatment with DHA significantly shortened the feeding latency (NMS + DHA: *n* = 10, *p* < 0.05 vs. NMS + NS; Figure 1H) and obviously increased the amount of food consumed during the experimental period in NMS rats (NMS + DHA: *n* = 10, *p* < 0.01 vs. NMS + NS; Figure 1I). Taken together, these results suggest that systemic administration of DHA can prevent anxiety‐like behavior induced by NMS.

### 3.4 Construction of DHA‐ASD intersection targets network

**FIGURE 2:**
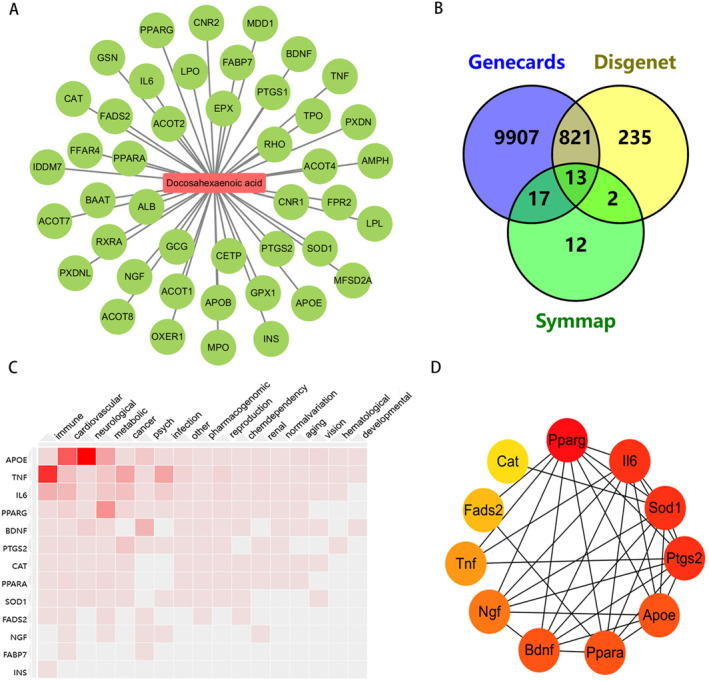
Identification of intersection targets shared by ASD and DHA. (A) Ingredient‐target network analysis. (B) Venn diagram of ASD and DHA target genes. (C) Hierarchical cluster analysis of Gene‐Disease Associations (GAD): correlation of disease about DHA target genes. (D) Network analyses of the 11 genes from Venn with STRING. DHA, docosahexaenoic acid.

In this study, we employed the SymMap database to find DHA targets and constructed a DHA‐target interaction network utilizing the Cytoscape v3.9.1 program (Figure 2A). The components of DHA are represented by green nodes, and each connecting line between a component and a target gene describes their interaction. We obtained ASD‐related gene targets by searching GeneCards and SymMap with the DisGeNET database and identified 13 overlapping genes (TNF, IL6, SOD1, CAT, PTGS2, BDNF, NGF, APOE, FABP7, FADS2, INS, PPARA, and PPARG) by searching for the intersection of disease targets and the aforementioned drug targets (Figure 2B). Therefore, we then analyzed these genes for disease‐related enrichment and displayed them in a heat map of the Harmonizome, the results showed that these genes are closely associated with neurological diseases (Figure 2C).

To establish a target network, we uploaded 13 overlapping genes shared between DHA and ASD to the STRING database to explore potential interactions among these targets (Figure 2D). Proteins were represented by network nodes, while PPI was represented by network connectivity. PPI was scored based on their degree using the cytosHubba plugin, with colors indicating high or low scores, such as red indicates high scores and yellow indicates low scores. It was found that 11 of these 13 targets have interactions.

### 3.5 Functional enrichment analysis

**FIGURE 3:**
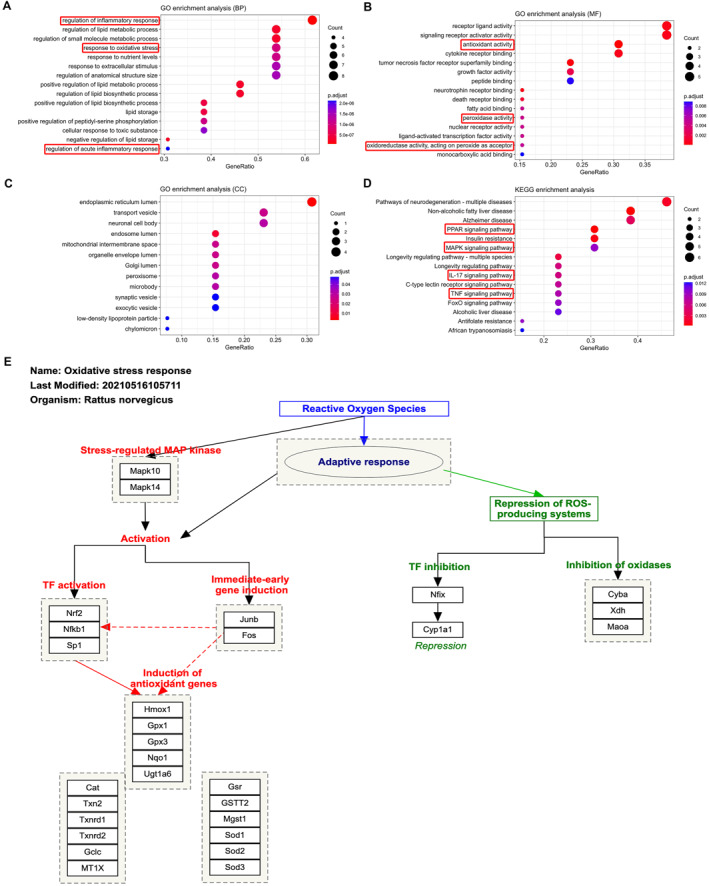
GO, KEGG enrichment and Wikipedia enrichment analysis result of the 13 overlapping genes. GO analysis of 13 overlapping genes showed (A) BP is mainly involved in the regulation of inflammatory response, response to oxidative stress, response to extracellular stimulus, and so on, (B) MF is mainly associated with signaling receptor activator activity, antioxidant activity, cytokine receptor binding, neurotrophic receptor binding, peroxidase activity, and so on, (C) CC is mainly associated with endoplasmic reticulum lumen, transport vesicle, neuronal cell body, synaptic vesicle, and so on. (D) KEGG analysis of 13 overlapping genes showed the most significantly enriched 15 pathways included PPAR signaling pathway, MAPK signaling pathway, IL‐17 signaling pathway and TNF signaling pathway. (E) Wikipedia enrichment analysis revealed that those 13 overlapping genes were enriched to oxidative stress response.

We performed GO and KEGG functional enrichment analyses of 13 genes using Hiplot to investigate the mechanism of action of DHA in ASD treatment. The GO analysis included 3 levels: BP, CC, and MF. The results of BP, MF and CC are shown in Figure 3A–C (bubble plots), respectively. The BP is mainly involved in the regulation of inflammatory response, response to oxidative stress, response to extracellular stimulus, and so on. MF is mainly associated with signaling receptor activator activity, antioxidant activity, cytokine receptor binding, neurotrophic receptor binding, peroxidase activity, and so on. CC is mainly associated with endoplasmic reticulum lumen, transport vesicle, neuronal cell body, synaptic vesicle, and so on. KEGG enrichment analysis result showed the 15 most significantly enriched pathways (Figure 3D) including PPAR signaling pathway, MAPK signaling pathway, IL‐17 signaling pathway, and TNF signaling pathway. Analysis using Wikipedia enrichment revealed that those 13 overlapping genes were enriched in oxidative stress response (Figure 3E). These results suggest that DHA may alleviate ASD through anti‐oxidative stress response, anti‐inflammatory, and modulating other pathways.

### 3.6 Molecular docking validation

**FIGURE 4:**
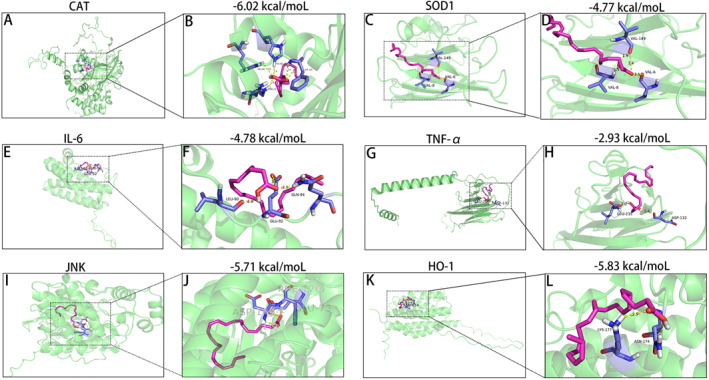
Molecular docking of DHA complexed with CAT, SOD1, IL‐6, TNF‐α, JNK and HO‐1 was analyzed using AutoDock software. (A, C, E, G, I, K) Molecular docking of DHA complexed with CAT, SOD1, IL‐6, TNF‐α, JNK and HO‐1. (B, D, F, H, J, L). The binding energy between DHA and CAT (−6.02 kcal/moL), SOD1 (−4.77 kcal/moL), IL‐6 (−4.78 kcal/mol), TNF‐α (−2.93 kcal/moL), JNK (−5.71 kcal/moL) or HO‐1 (−5.83 kcal/mol) was low, and the active pocket formed by CAT, SOD1, IL‐6, TNF‐α, JNK and HO‐1 was relatively stable, respectively. DHA, docosahexaenoic acid.

By estimating the binding energy, molecular docking was used to simulate the interaction between ligand and receptor and predict the affinity. KEGG and Wikipedia enrichment analyses showed that CAT (Alphafold: P04762), SOD1 (Uniprot *Homo sapiens*), IL‐6 (Alphafold: P20607) and TNF‐α (Alphafold: P16599) are involved in oxidative stress and inflammatory response‐related pathways. In the Wikipedia enrichment analysis, we saw the c‐JUN molecule in the JNK signaling pathway, and it has been demonstrated that c‐JUN can probably be a candidate gene for ASD diagnosis.^44^ The activation of JNK signaling pathway mediates the cellular inflammatory response and oxidative stress‐related diseases.^45^ Therefore, we speculated whether DHA could act on JNK molecule. JNK (UniProt: Q6P727) and oxidative stress pathway downstream protein HO‐1 (Alphafold: P06762) were selected for subsequent analysis. The binding energy between DHA and CAT (−6.02 kcal/moL), SOD1 (−4.77 kcal/moL), IL‐6 (−4.78 kcal/mol), TNF‐α (−2.93 kcal/moL), JNK (−5.71 kcal/moL) and HO‐1 (−5.83 kcal/moL) were low, and the active pocket formed by CAT, SOD1, IL‐6, TNF‐α, JNK and HO‐1 were relatively stable (Figure 4), indicating that DHA binds to CAT, SOD1, IL‐6, TNF‐α, JNK and HO‐1 directly.

### 3.7 Experimental evaluation

#### 3.7.1 DHA inhibits JNK signaling pathway activation in NMS rats

**FIGURE 5:**
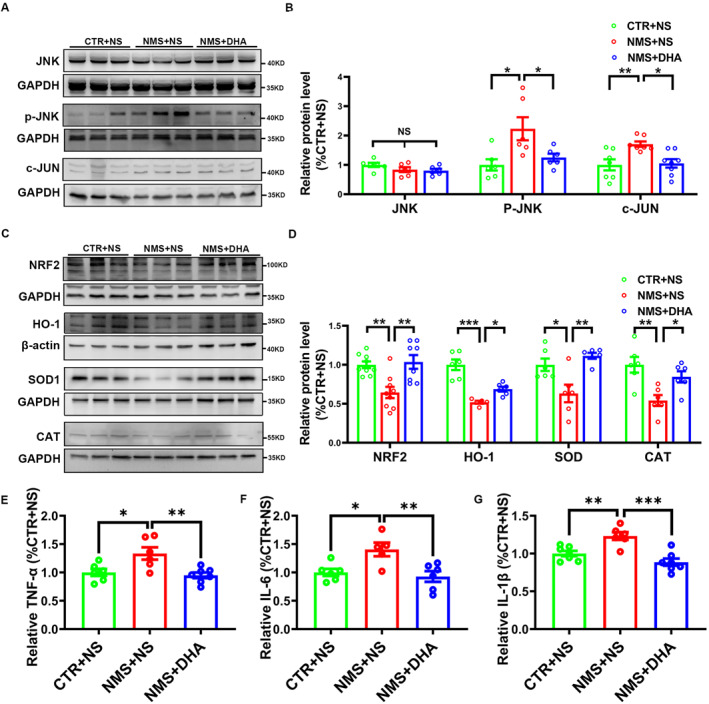
DHA suppresses JNK activation and inhibits oxidative stress and inflammatory response. Western blotting results showed: (A‐B) DHA treatment significantly inhibited the elevation of protein levels p‐JNK and c‐JUN induced by NMS, while not affecting the expression of JNK. (C‐D) DHA treatment significantly upregulated the levels of NRF2, HO‐1, SOD1 and CAT in NMS rats. (E‐G) ELISA assays showed DHA treatment reduced the release of pro‐inflammatory cytokines, such as TNF‐α (E), IL‐6 (F), and IL‐1β (G) in NMS rats. The results were presented as the mean ± SEM. DHA, docosahexaenoic acid; ELISA, enzyme‐linked immunosorbent assay; NMS, neonatal maternal separation. *p < 0.05, **p < 0.01, ***p < 0.001.

As oxidative stress and inflammatory responses are crucial factors in the pathological process of ASD, DHA exhibits prominent anti‐oxidative and anti‐inflammatory properties, and the activation of JNK signaling pathway mediates the cellular inflammatory response and oxidative stress‐related diseases.^7^, ^8^, ^9^, ^14^ Targeting JNK in neurons and inhibiting its activation have been reported as a treatment for neurological diseases.^46^ A recent study in an ischemic brain injury model confirms DHA exerts neuroprotective effects by inhibiting JNK abnormal phosphorylation.^47^ Research also reported DHA decreased JNK activation in microglia and pro‐inflammatory factor release, alleviating neuropathic pain.^48^ Furthermore, our network pharmacology and molecular docking results suggest that DHA may exert protective effects in ASD by suppressing the JNK pathway, thereby exerting antioxidant and anti‐inflammatory effects. Therefore, here we want to verify whether DHA exhibits the potential to suppress JNK activation and inhibit oxidative stress and inflammatory response, thereby improving autistic‐like behaviors induced by NMS in rats. Initially, we detected the expression levels of JNK, p‐JNK, and c‐JUN proteins in PFC. As shown in Figure 5, although there was no significant difference in the total expression level of JNK (CTR + NS: *n* = 6; NMS + NS: *n* = 6, *p* > 0.05 vs. CTR + NS; NMS + DHA: *n* = 6, *p* > 0.05 vs. NMS + NS; Figure 5A,B) and in PFC proteins of rats in each group, the active form of p‐JNK (CTR + NS: *n* = 6; NMS + NS: *n* = 6, *p* < 0.05 vs. CTR + NS; Figure 5A,B) and its downstream protein c‐JUN (CTR + NS: *n* = 7; NMS + NS: *n* = 7, *p* < 0.01 vs. CTR + NS; Figure 5A,B) were significantly increased in the NMS + NS group. Interestingly, the increased protein levels of p‐JNK and c‐JUN were significantly reversed by treatment with DHA in NMS rats (For p‐JNK: NMS + DHA: *n* = 6, *p* < 0.05 vs. NMS + NS; For c‐JUN: NMS + DHA: *n* = 8, *p* < 0.05 vs. NMS + NS, Figure 5A,B). These results suggest DHA inhibits JNK signaling pathway activation induced by NMS.

#### 3.7.2 DHA inhibits oxidative stress in NMS rats

Next, we detected the expression of oxidative stress‐related proteins. As shown in Figure 5, compared with CTR + NS group, the expressions of NRF2 (CTR + NS: *n* = 6; NMS + NS: *n* = 6, *p* < 0.01 vs. CTR + NS; Figure 5C,D), HO‐1 (CTR + NS: *n* = 6; NMS + NS: *n* = 6, *p* < 0.001 vs. CTR + NS; Figure 5C,D), SOD1 (CTR + NS: *n* = 6; NMS + NS: *n* = 6, *p* < 0.05 vs. CTR + NS; Figure 5C,D) and CAT (CTR + NS: *n* = 6; NMS + NS: *n* = 6, *p* < 0.01 vs. CTR + NS; Figure 5C,D) in the PFC were significantly decreased in NMS + NS group. Notably, DHA treatment effectively restored the downregulation of NRF2, HO‐1, SOD1, and CAT triggered by NMS in the PFC of brain, as shown by NRF2 (NMS + DHA: *n* = 6, *p* < 0.01 vs. NMS + NS; Figure 5C,D), HO‐1 (NMS + DHA: *n* = 6, *p* < 0.05 vs. NMS + NS; Figure 5C,D), SOD1 (NMS + DHA: *n* = 6, *p* < 0.01 vs. NMS + NS; Figure 5C,D) and CAT (NMS + DHA: *n* = 6, *p* < 0.05 vs. NMS + NS; Figure 5C,D). These results suggest DHA inhibits oxidative stress activation induced by NMS.

#### 3.7.3 DHA reduces inflammatory cytokines production in NMS rats

To further explore the protective mechanism of DHA in NMS rats, we measured the expression of inflammation cytokines such as TNF‐α, IL‐6, and IL‐1β. The results showed that NMS caused a significant increase in the secretion of TNF‐α, IL‐6, and IL‐1β (For TNF‐α: CTR + NS: *n* = 6; NMS + NS: *n* = 6, *p* < 0.05 vs. CTR + NS; Figure 5E; For IL‐6: CTR + NS: *n* = 6; NMS + NS: *n* = 5, *p* < 0.05 vs. CTR + NS; Figure 5F; For IL‐1β: CTR + NS: *n* = 7; NMS + NS: *n* = 6, *p* < 0.01 vs. CTR + NS; Figure 5G). Notably, treatment with DHA dramatically rescued the increase of TNF‐α, IL‐6 and IL‐1β induced by NMS in the brain (For TNF‐α: NMS + DHA: *n* = 7, *p < *0.01 vs. NMS + NS; Figure 5E; For IL‐6: NMS + DHA: *n* = 6, *p* < 0.01 vs. NMS + NS; Figure 5F; For IL‐1β: NMS + DHA: *n* = 7, *p* < 0.001 vs. NMS + NS; Figure 5G). Together, these results suggest DHA reduces inflammatory cytokines production induced by NMS.

## 4 DISCUSSION

In recent years, the study of ASD has attracted much attention. The pathophysiologic mechanisms of ASD are complex, with many influencing factors, leading to many challenges in the process of treating ASD. Much evidence suggests that inflammation and oxidative stress are important etiologic factors in ASD.^49^, ^50^ The emergence of network pharmacology offers insights into the intricate interactions between compounds and their targets.^51^ In the present study, we employed network pharmacology methods to predict effective molecular targets and underlying mechanisms of DHA in the treatment of ASD. Subsequently, biochemical assays were conducted to further investigate and identify the potential mechanisms of DHA in treating ASD.

DHA, a vital polyunsaturated fatty acid indispensable for human health, has garnered significant attention in past research studies, particularly in exploring its role in neurodevelopment and memory enhancement.^15^, ^52^, ^53^ It has been found that DHA accumulates during the most active periods of brain growth, neurogenesis and synaptogenesis, thus promoting synaptic growth, synapse formation, and increased synaptic activity.^24^, ^54^ DHA also maintains the long‐term potentiation and enhances signaling between synapses, which is essential for maintaining learning and memory functions.^55^ In addition, DHA enrichment in the brain has anti‐apoptotic effects and protects neurons in unfavorable environments.^56^ For children suffering from developmental and behavioral disorders who have low blood levels of polyunsaturated fatty acids, supplementation with DHA can be beneficial in enhancing their learning capabilities and improving abnormal behavior.^19^ In the present study, treatment with DHA from the first day after birth can significantly ameliorate autistic‐like behaviors caused by NMS in rats, as shown in Figure 1.

Increasing evidence from various studies has found that JNK is involved in numerous signaling pathways and holds a pivotal role in the development and progression of ASD.^57^, ^58^, ^59^ JNK, a factor of MAPK signaling pathway, can be activated by a variety of environmental stimuli. c‐JUN, a factor of JNK signaling pathway, causes neuroinflammation generation through promoting the release of pro‐inflammatory mediators in the central nervous system (CNS).^60^ A recent study found that c‐JUN could potentially serve as a diagnostic marker for ASD.^44^ This indicates that modulating the expression of the c‐JUN gene may hold promise for therapeutically beneficial outcomes in ASD treatment. Research studies have found that DHA exists the ability to inhibit the JNK signaling pathway and suppress inflammatory response.^21^ Based on the available evidence, we postulated that the therapeutic potential of DHA in treating ASD might be linked to its modulation of the JNK/c‐JUN signaling pathway. First, molecular docking analysis showed that DHA had a favorable binding affinity for JNK (Figure 4). Second, our in vivo study showed that DHA could obviously inhibit the phosphorylation of JNK and the elevated level of c‐JUN induced by NMS (Figure 5). Since JNK pathway cause neuroinflammation generation by promoting the release of pro‐inflammatory mediators in the CNS,^60^ including TNF‐α, IL‐6, and IL‐1β, which are some classic pro‐inflammatory mediators, we then detected the levels of TNF‐α, IL‐6, and IL‐1β, as shown in Figure 5. DHA could evidently decrease the elevated level of TNF‐α, IL‐6, and IL‐1β induced by NMS. These results demonstrated that the therapeutic effect of DHA in treating ASD is associated with its ability to inhibit the JNK/c‐JUN signaling pathway and suppress the release of pro‐inflammatory cytokines.

Oxidative stress disorders are common drivers of the pathogenesis of neurological diseases such as ASD. Many reports have found a significant increase of oxidative stress markers in the brain and peripheral circulation in children with ASD, with levels correlating with the extent of the disease.^7^, ^8^, ^9^ Furthermore, antioxidant supplementation can be helpful for some children with autism.^10^ Also, if the transcription of NRF2, a key regulator of the cellular oxidative stress response pathway, is effectively regulated to maintain a stable expression level in pathological conditions, it can promote the expression of antioxidant proteins.^61^ In addition, research studies have revealed that DHA exhibits inhibitory effects on oxidative stress and concurrently upregulates NRF2.^62^, ^63^ In the present study, our network pharmacological results showed that the intersection of DHA with ASD target genes is mainly involved in oxidative stress‐related pathways (Figure 3). NRF2, HO‐1, CAT and SOD1 are directly related to oxidative stress pathways; therefore, disorders of these genes may lead to ASD. Moreover, we further conducted biochemical assays and found that NMS triggered oxidative stress reactions and treatment with DHA significantly suppressed oxidative stress, as shown in Figure 5.

In summary, through a comprehensive approach encompassing network pharmacology, molecular docking, and biochemical assays, we predicted and validated the underlying mechanism of how DHA alleviates autistic‐like behaviors. Our findings indicate that DHA has the capacity to attenuate JNK activation, mitigate oxidative stress, and quell inflammatory responses, thereby improving autistic‐like behaviors triggered by NMS in rats. These results suggest that DHA holds promise as a potential therapeutic agent for the treatment of ASD. However, further research is needed to fully understand the underlying mechanisms of DHA in ASD. Also, there are two drawbacks to this study. On the one hand, our study only focuses on the effect of offspring's behavior for a short period caused by NMS, but this effect is likely to be lifelong. Therefore, more relevant experiments are needed to observe it. Second, a larger database of target genes is needed, which will improve the accuracy of the results of network pharmacology analysis. These shortcomings will be improved in subsequent studies.

## 5 CONCLUSION

DHA plays a crucial role in the prevention and treatment of ASD. In the present study, the key targets of DHA for ASD treatment were screened for the first time using network pharmacological analysis and molecular docking. The results suggest that DHA improves autistic‐like behaviors induced by NMS in rats by suppressing JNK activation, mitigating oxidative stress, and quelling inflammatory responses. This study not only establishes a foundation for future investigations into the therapeutic mechanisms of DHA in treating ASD but also underscores the urgent necessity for such innovative approaches in the realm of drug development for related conditions.

## AUTHOR CONTRIBUTIONS

Chunfang Dai and Zhifang Dong conceived the study and wrote the manuscript. Boqing Xu and Hao Yuan performed network pharmacology analysis and molecular docking. Boqing Xu and Xiaohuan Li performed behavioral studies. Boqing Xu, Hao Yuan and Qingyang Yu performed biochemical assays. All authors read and approved the final manuscript.

## CONFLICT OF INTEREST STATEMENT

The authors declare no conflict of interests.

## ETHICS STATEMENT

All animal experiments were performed in accordance with the Chongqing Science and Technology Commission guidelines and approved by the Animal Ethics Committee of Children's Hospital of Chongqing Medical University (No.CHCMU‐IACUC20220323015).

## Data Availability

The data that support the findings of this study are available from the corresponding author upon reasonable request.
